# Return to work following diagnosis of low-grade glioma: A nationwide matched cohort study

**DOI:** 10.1212/WNL.0000000000009982

**Published:** 2020-08-18

**Authors:** Isabelle Rydén, Louise Carstam, Sasha Gulati, Anja Smits, Katharina S. Sunnerhagen, Per Hellström, Roger Henriksson, Jiri Bartek, Øyvind Salvesen, Asgeir Store Jakola

**Affiliations:** From the Section of Clinical Neuroscience, Institute of Neuroscience and Physiology (I.R., L.C., A.S., K.S.S., P.H., A.S.J.), University of Gothenburg, Sahlgrenska Academy; Departments of Neurology (I.R., A.S., P.H.) and Neurosurgery (L.C., A.S.J.), Sahlgrenska University Hospital, Gothenburg, Sweden; Department of Neurosurgery (S.G., A.S.J.), St. Olavs University Hospital HF; Institute of Neuroscience (S.G.) and Department of Public Health and Nursing (Ø.S.), Norwegian University of Science and Technology, Trondheim, Norway; Department of Neuroscience (A.S.), Uppsala University; Department of Radiation Sciences & Oncology (R.H.), University of Umeå; Department of Neurosurgery (J.B.), Karolinska University Hospital; Departments of Neuroscience and Medicine (J.B.), Karolinska Institutet, Stockholm, Sweden; and Department of Neurosurgery (J.B.), Copenhagen University Hospital Rigshospitalet, Denmark.

## Abstract

**Objective:**

Return-to-work (RTW) following diagnosis of infiltrative low-grade gliomas is unknown.

**Methods:**

Swedish patients with histopathologic verified WHO grade II diffuse glioma diagnosed between 2005 and 2015 were included. Data were acquired from several Swedish registries. A total of 381 patients aged 18–60 were eligible. A matched control population (n = 1,900) was acquired. Individual data on sick leave, compensations, comorbidity, and treatments assigned were assessed. Predictors were explored using multivariable logistic regression.

**Results:**

One year before surgery/index date, 88% of cases were working, compared to 91% of controls. The proportion of controls working remained constant, while patients had a rapid increase in sick leave approximately 6 months prior to surgery. After 1 and 2 years, respectively, 52% and 63% of the patients were working. Predictors for no RTW after 1 year were previous sick leave (odds ratio [OR] 0.92, 95% confidence interval [CI] 0.88–0.96, *p* < 0.001), older age (OR 0.96, 95% CI 0.94–0.99, *p* = 0.005), and lower functional level (OR 0.64 95% CI, 0.45–0.91 *p* = 0.01). Patients receiving adjuvant treatment were less likely to RTW within the first year. At 2 years, biopsy (as opposed to resection), female sex, and comorbidity were also unfavorable, while age and adjuvant treatment were no longer significant.

**Conclusions:**

Approximately half of patients RTW within the first year. Lower functional status, previous sick leave, older age, and adjuvant treatment were risk factors for no RTW at 1 year after surgery. Female sex, comorbidity, and biopsy only were also unfavorable for RTW at 2 years.

Adult supratentorial WHO grade II diffuse low-grade gliomas (LGGs) are slow-growing primary brain tumors. LGG typically affect young adults in the middle of their life and career, and for the majority the disease presents with seizures only.^1,2^ The slow growth allows for functional reorganization and patients usually have no visible or only minor functional disabilities at disease onset.^3^

With active surgical and oncologic treatment, the median survival now exceeds 10 years following diagnosis.^4–7^ It is frequently emphasized that not only longevity, but also the patients' overall functioning and quality of life are important.^8^ Although permanent neurologic deficits due to treatment are less common,^9^ patients report compromised health-related quality of life.^10,11^ Similar to patients with other types of cancers, patients with LGG report problems with social functioning and fatigue, but with the additional burden of seizures and cognitive complaints.^10^ Both the disease and the treatment may cause absence from work, but little is known concerning return to work (RTW) in patients with LGG.

RTW constitutes an important part of getting back to a normal life.^12–14^ Being able to work again is often a sign of successful rehabilitation and improved quality of life.^15^ Although patients with LGG are frequently concerned about RTW in patient consultation prior to treatment decisions, RTW is an understudied aspect. Work status in patients with LGG has only been reported in smaller series.^16–18^

The aim of this study was to study patterns of sick leave and explored predictors for RTW among patients with LGG in Sweden and compare this to a matched control group.

## Methods

We used data from nationwide Swedish registries. Linking of registries was possible through the unique personal identification numbers for Swedish citizens. The used registries are described below, and definitions of variables derived from the registries are described in detail in table 1*.*

**Table 1:**
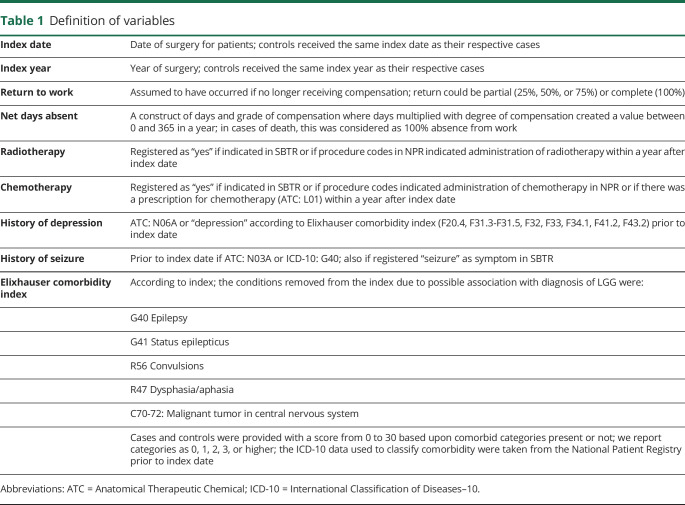
Definition of variables

### Swedish Brain Tumor Registry

The national Swedish Brain Tumor Registry (SBTR) is a regionally based registry of adult patients diagnosed with brain tumors carrying detailed information on tumor and patient characteristics. The level of coverage from the different regions has varied somewhat over time. In our study, a minimum registration rate of 80% was required to be included in the analysis at any given year for each region to provide representative population-based data. Registration rate was defined as the percentage of diagnoses in the SBTR that corresponds to diagnoses reported to the compulsory National Cancer Registry. Further details on the SBTR and definition of clinical variables in patients with LGG are available through our earlier work.^19,20^ Data from SBTR was accessed October 21, 2016.

### Statistics Sweden

Statistics Sweden (www.scb.se) is a government agency responsible for official and objective statistics for general information, investigation, and research in Sweden. We extracted data on education and disposable income. Educational level was graded according to the Swedish nomenclature for education.^21^ Level of education was divided into 2 groups: basic to high school (SUN2000 grade 1 through 4) and higher education (SUN2000 grade 5 through 7). A matched cohort of 5 individuals for each case was obtained, with year of birth, sex, municipality of residence, and educational level used as matching criteria. All controls were unique. For 9 cases, the amount of controls was incomplete. Data from Statistics Sweden were accessed June 26, 2017.

### Swedish Social Insurance Agency

The Social Insurance Agency (SIA) is a Swedish government agency that provides financial security in the event of illness. In Sweden, the employer provides the payment for the first 2 weeks of sick leave. Thereafter the SIA is responsible for the sickness benefit. SIA is responsible for holding official statistics on sick leave (temporary compensation) and disability compensation (for longer-lasting and permanent compensations, later referred to as permanent sick leave). Information provided was time periods with compensation (including the first 2 weeks reimbursed by the employer), type of compensation, and grade of compensation. The data from SIA were accessed January 31, 2018.

### National Board of Health and Welfare

The National Board of Health and Welfare (NBHW) is the government agency responsible for developing statistics about health care in different registries. From the National Patient Registry (NPR), we received data on days of inpatient and outpatient visits, including diagnostic and procedural codes in the 2003–2016 period. Since 2001, this registry has been subject to mandatory reporting from both private and public hospitals but does not include primary care contacts. The NPR thus contains information about all contact with specialist health care with diagnoses coded according to ICD-10. The ICD-10 codes were used to classify comorbidity according to the Elixhauser comorbidity index.^22,23^ Underreporting in the NPR has been estimated to be less than 1% according to the NBHW (www.socialstyrelsen.se). The national prescription registry was established July 1, 2005, with mandatory registration from start. From the prescription registry, we received information concerning type of drug according to the Anatomical Therapeutic Chemical classification system and date of dispensing in the period 1 year prior to index year and 2 years after index year, although limited by the registry being established in mid-2005. In this study, we used information on any chemotherapeutic (L01), antiepileptic (N03A), and antidepressant (N06A) drug prescription. The registries under NBHW were accessed January 8, 2018.

### Patient selection

**Figure 1:**
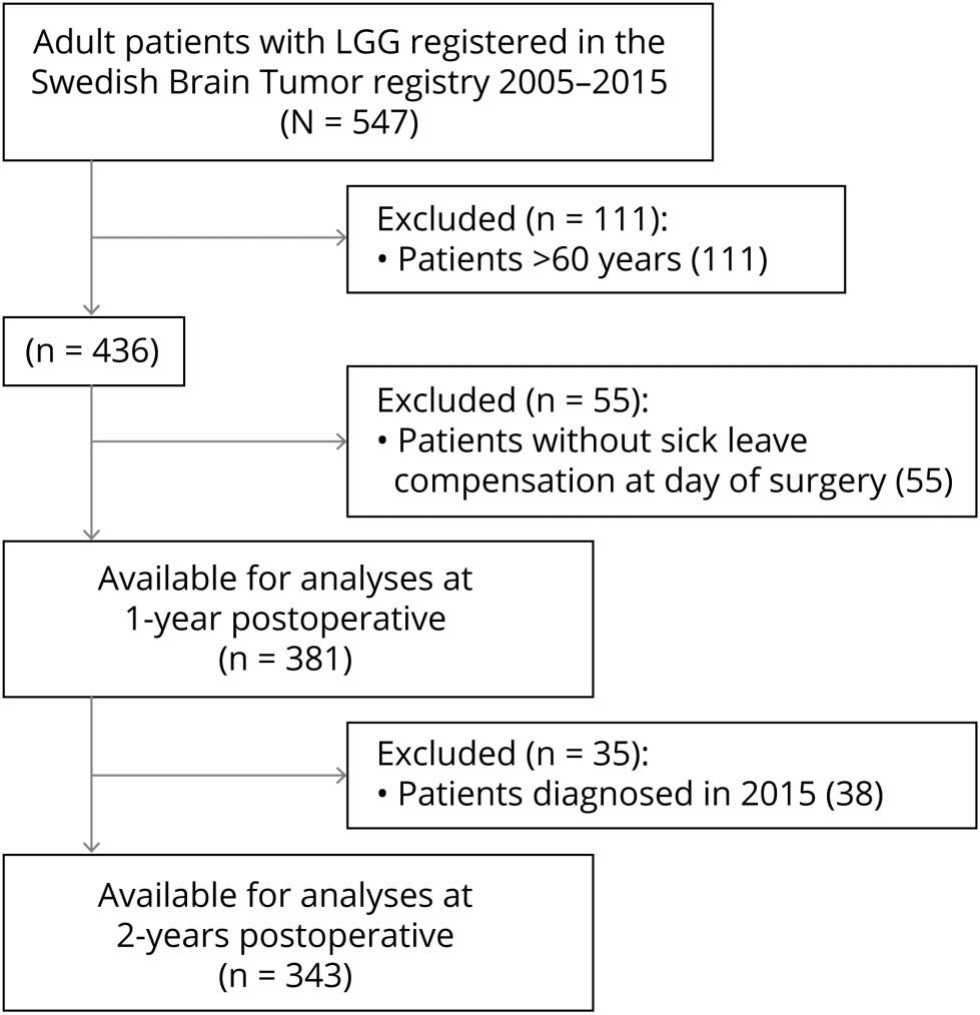
Flow chart of patient selection LGG = low-grade glioma.

Using the SBTR, we identified 547 adults (≥18 years) between 2005 and 2015 with a first-time diagnosis of supratentorial hemispheric diffuse LGG, defined as WHO grade II astrocytoma, oligoastrocytoma, or oligodendroglioma according to the 2007 WHO classification of brain tumors.^24^ Patients with radiologically suspected LGG only were not included in the present study. Since our focus was RTW, we only included patients between 18 and 60 years (n = 436), as done by others.^25^ Further, some of these patients may not have been in activity related to work and consequently not entitled to compensation from the SIA. For this reason, we only included patients who received some form of compensation for absence from work at the day of surgery (n = 381) and their respective controls (n = 1,900). For analyses at 2 years following surgery, we selected patients between 2005 and 2014 to ensure adequate follow-up (n = 343). The selection process is summarized in figure 1*.*

### Statistics

Data from the different registries were imported into corresponding tables in a mySQL (Oracle). Temporary and permanent sick leave compensation were combined for each individual using Python version 2.7 (Python Software Foundation). Other data derivations were done using mySQL. R Statistical Software version 3.1 was used for statistical analyses.

Continuous variables were summarized using the median and first and third quartiles and compared between cases and controls using the Mann-Whitney *U* test. Categorical variables were summarized using counts and proportions and compared between cases and controls using the Fisher exact test.

Univariable and multivariable logistic regression analysis were done to examine predictors of RTW and independent predictors of RTW, respectively. In the regression model, RTW at 1 year was defined as any work-related activity (25%–100%) at day 365 postoperative. We only included baseline factors in the model. Covariates in the regression models were chosen based upon presumed clinical relevance. Among demographic variables, we included age and sex. Socioeconomic variables included disposable income and educational level at index year, and net days absence 365 days prior to index date. We considered the possibility that patients had lower disposable income the index year than the year prior to the index year; however, since we observed the opposite, we used the index year in our calculations. Finally, clinical variables included functional status (WHO performance status), tumor size (<4, 4–6, >6 cm), history of seizures (no/yes), history of depression (no/yes), and other comorbidity using Elixhauser comorbidity index.^22,23^ In a sensitivity analysis of any work-related activity at 365 days following index date, we also included factors related to postoperative treatment.

For each day from 365 days prior to index date until 365 days after index date, counts of persons without sick leave compensation, of persons with partial compensation, of persons with full compensation, and of deceased persons were computed and displayed in stacked graphs for cases and controls separately.

All tests were 2-sided and we considered a *p* value <0.05 to be significant.

### Standard protocol approvals, registrations, and patient consents

The Regional Ethical Review Board in Gothenburg approved this study (Dnr: 702-16).

### Data availability

Due to restrictions from the registry holders, raw data cannot be shared.

## Results

### Demographic data

In the included patients with LGG, the mean age was 41.4 years and 55% were male. Details concerning baseline and treatment characteristics for patients with LGG are presented in table 2.

**Table 2:**
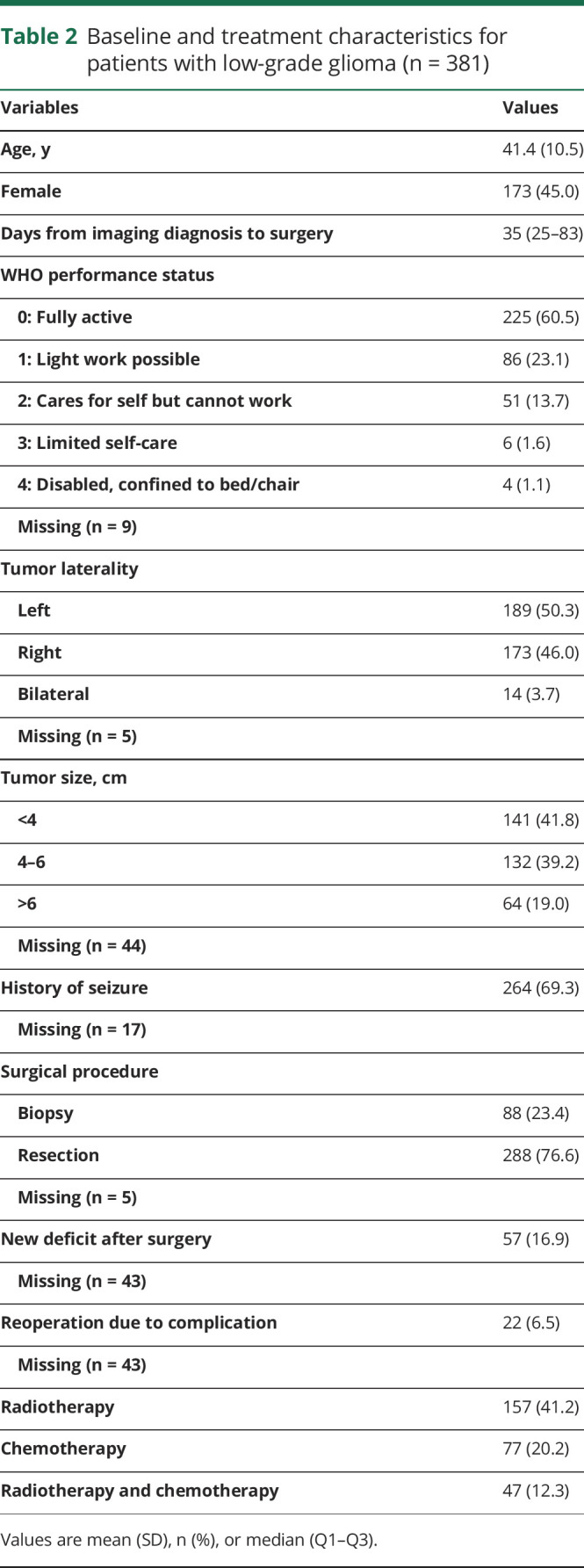
Baseline and treatment characteristics for patients with low-grade glioma (n = 381)

### Sick leave compensation

The socioeconomic characteristics and comorbidity of patients and controls are presented in table 3. The median net days absent were 30 days for patients and 0 days for controls the year prior to index date (*p* < 0.001). The median net days absent were 345 days for patients and 0 days for controls the first year following index date (*p* < 0.001).

**Table 3:**
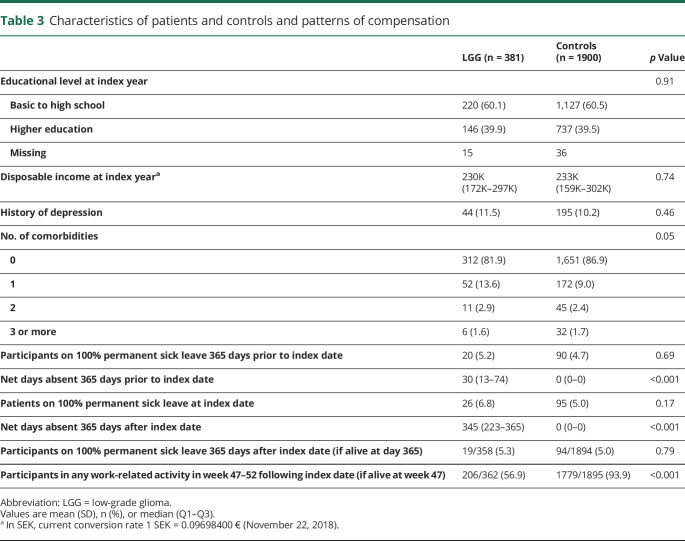
Characteristics of patients and controls and patterns of compensation

**Figure 2:**
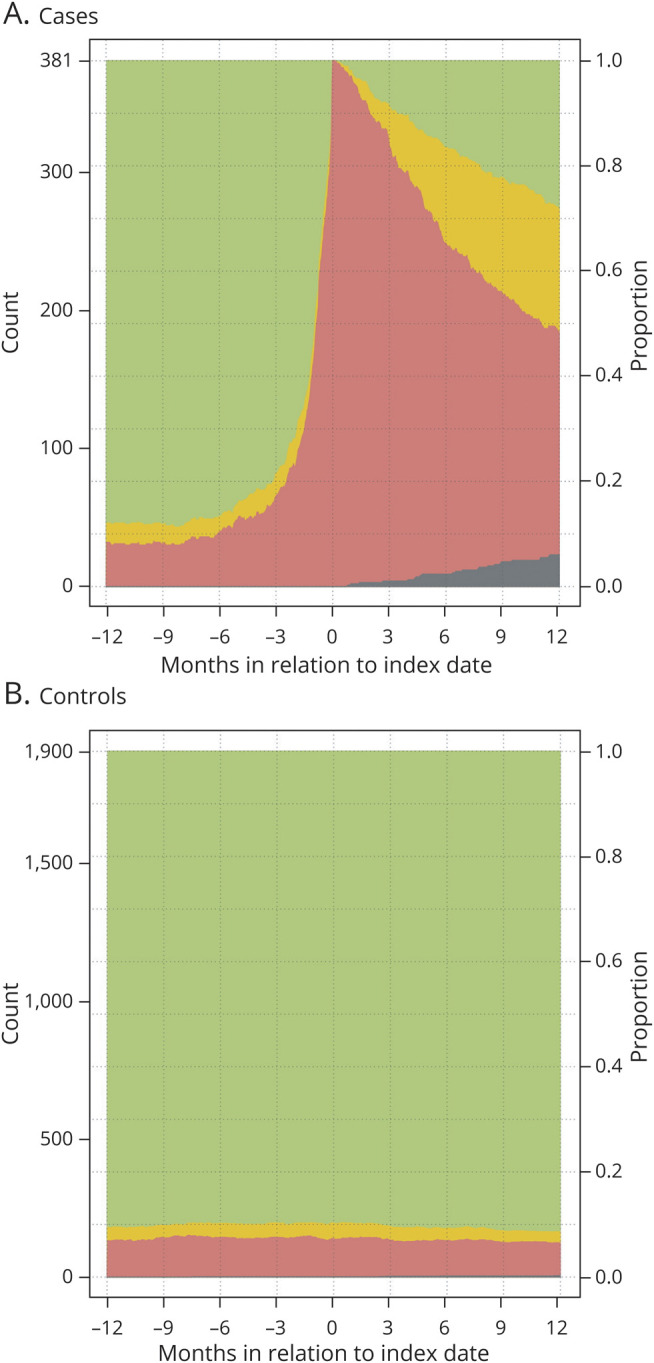
Sick leave compensation over time (12 months after index date) (A) Patients with low-grade glioma (n = 381) and (B) controls (n = 1,900) without sick leave compensation (green), with partial compensation (yellow), and with full compensation (red) 1 year prior to and 1 year following the index date (date of primary surgery) (n = 381). The dark gray stack at the bottom represents deceased patients.

The proportions of patients and controls obtaining compensation the year prior to index date and 1 year following index date are presented in figure 2. At 1 year following index date, the RTW rate among patients was 52% with 28% working full time, while 6% were deceased.

**Figure 3:**
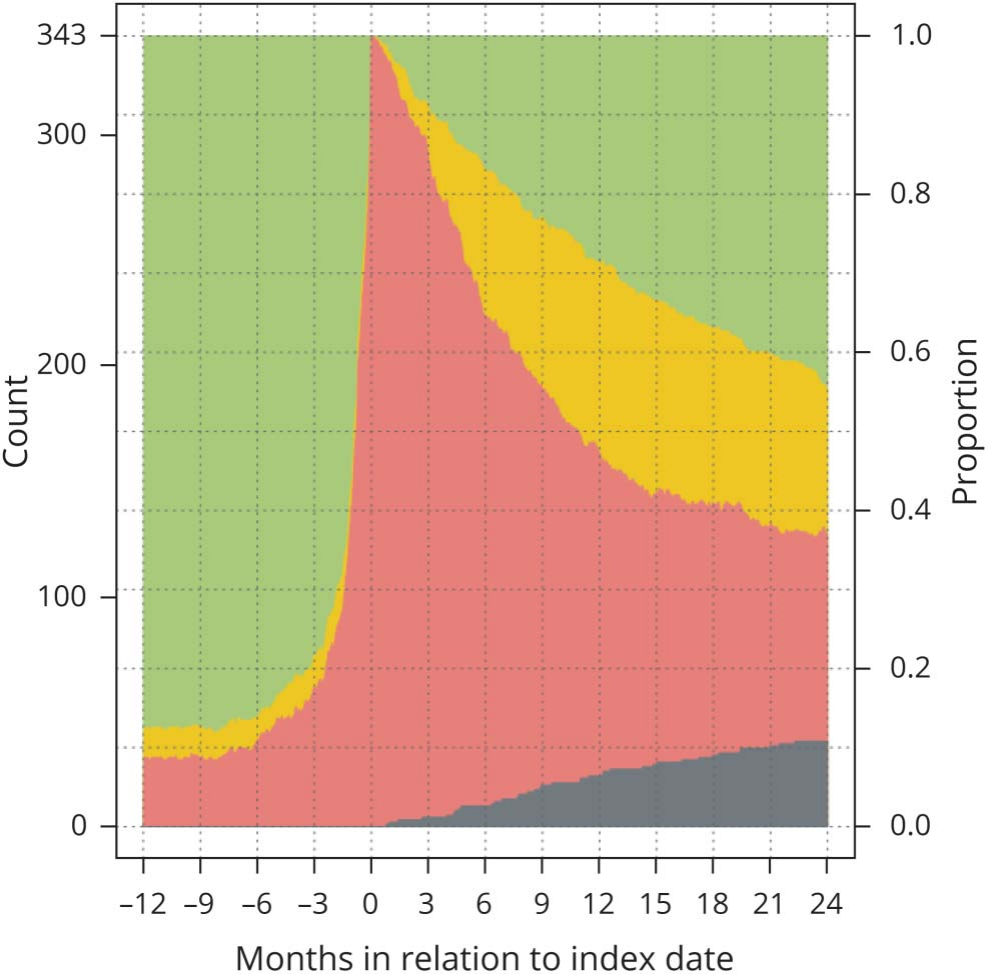
Sick leave compensation over time (24 months after index date) Patients with low-grade glioma without sick leave compensation (green), with partial compensation (yellow), and with full compensation (red) 1 year prior to and 2 years following the index date (date of primary surgery), including only patients with 2 years follow-up data available (n = 343). The dark gray stack at the bottom represents deceased patients.

**Figure 4:**
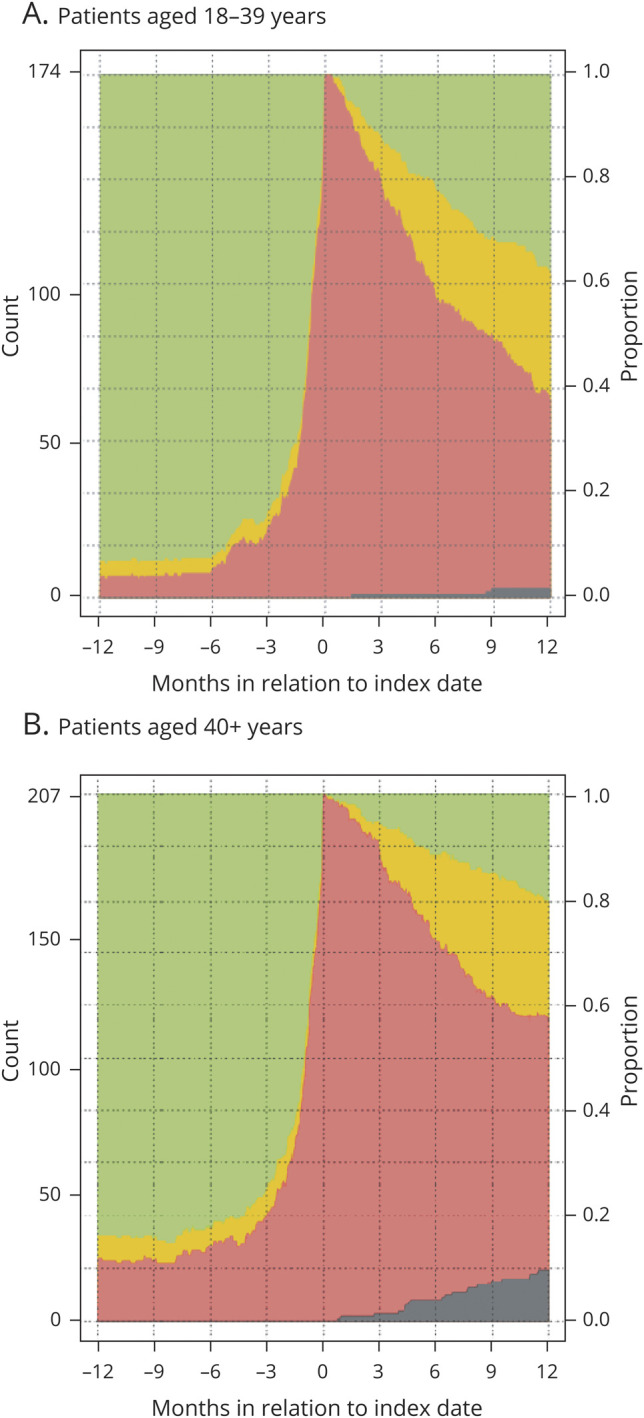
Sick leave compensation over time in relation to age (A) Patients aged 18–39 years. (B) Patients aged 40+ years.

**Figure 5:**
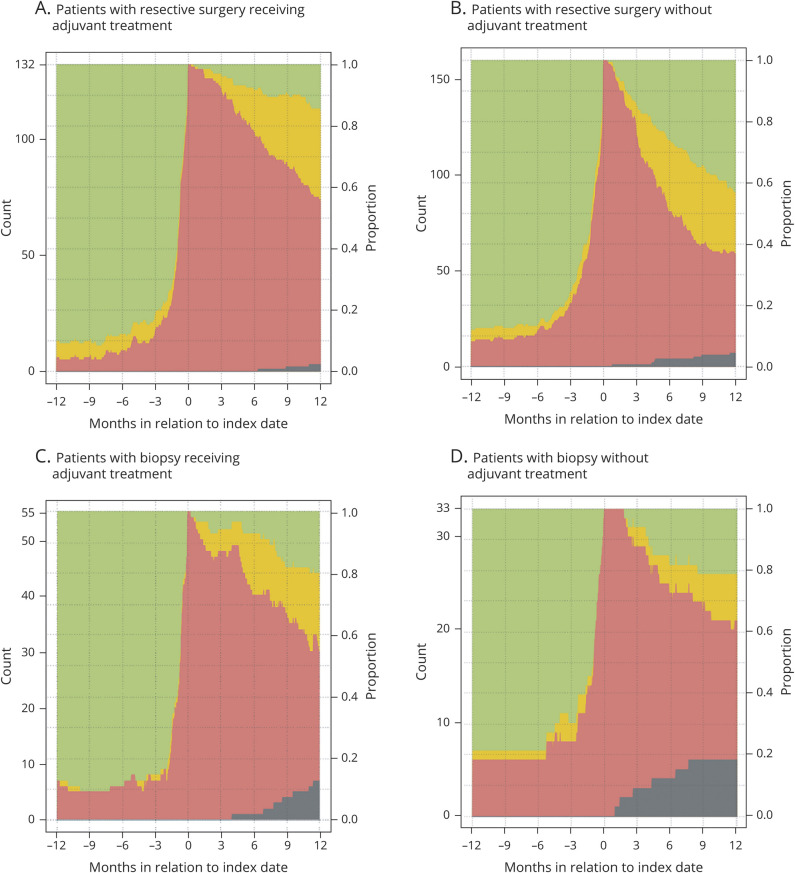
Sick leave compensation over time in relation to treatment combinations (A) Patients with resective surgery receiving adjuvant treatment. (B) Patients with resective surgery without adjuvant treatment. (C) Patients with biopsy receiving adjuvant treatment. (D) Patients with biopsy without adjuvant treatment.

Figure 3 demonstrates the proportions with compensation during the following 2 years after index date. At 2 years, the RTW rate among patients was 63% with 45% working full time, while 11% were deceased. In figure 4, we provide work status in relation to age and in figure 5 treatment combinations in relation to work status is shown 1 year before and after surgery. Finally, the same visualizations are provided for additional subgroups (sex, side, size, and functional status) (figures e-1 to e-4; doi:10.5061/dryad.2fqz612kf).

### Predictors of RTW

We explored predictors of RTW as outlined in table 4. At 1 year after surgery, previous absence from work, older age, lower functional level, and earlier index year were unfavorable predictive factors for RTW.

**Table 4:**
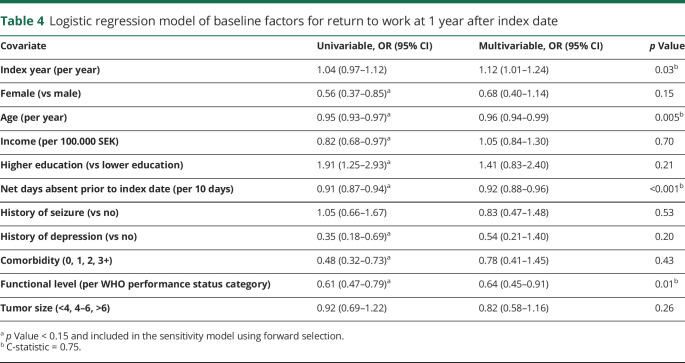
Logistic regression model of baseline factors for return to work at 1 year after index date

In a sensitivity analysis using forward selection, female sex (odds ratio [OR] 0.60, 95% confidence interval [CI] 0.37–0.97, *p* = 0.04) replaced index year. The other significant factors were age (OR 0.96%, 95% 0.94–0.98, *p* < 0.001), net days absent (OR per 10 days 0.92, 95% CI 0.88–0.96, *p* < 0.001), and functional status (OR 0.71%, 95% 0.52–0.96, *p* = 0.02). C-statistic for this model was 0.75. We also intended to explore RTW in week 47–52 (a more relaxed criteria) in another sensitivity analysis, but the difference in this measure compared to those returning within 365 days was clinically irrelevant (table 3) with only 9 additional patients when applying the more relaxed criteria. Instead, we created a sensitivity analysis where we also included tumor laterality (right vs left, excluded bilateral), treatment variables (resection vs biopsy, chemotherapy, radiotherapy), reoperation due to complications, and new neurologic deficits. In this analysis, net days absent (OR per 10 days 0.92, 95% CI 0.87–0.96, *p* = 0.02) together with functional level (OR 0.64, 0.44–9.94 *p* = 0.02) remained significant predictors for RTW. In this model, chemotherapy (OR 0.49, 95% CI 0.24–1.00, *p* = 0.05) and radiotherapy (OR 0.53, 95% CI 0.29–0.95, *p* = 0.03) were unfavorable for RTW.

We hypothesized that baseline factors (table 2) could be more important for RTW at 2 years following index date. Predictors for RTW at 2 years were sought in a regression model identical to the one presented in table 4. This analysis demonstrated that functional level, female sex, older age, and previous absence from work were negative predictors for RTW at 2 years (table e-1; doi:10.5061/dryad.2fqz612kf). In line with the analyses made at 1 year, we did a similar sensitivity analysis including the postoperative variables. By doing so, age was no longer significant (OR 0.97, 95% CI 0.94–1.00, *p* = 0.08) and replaced by comorbidity (OR 0.42, 95% CI 0.18–0.98, *p* = 0.05). The model confirmed the parameters female sex (OR 0.40, 95% CI 0.21–0.77, *p* = 0.007), functional level (OR 0.47, 95% CI 0.31–0.71, *p* < 0.001), and net days absent before index date (OR per 10 days 0.95, 95% CI 0.91–0.99, *p* = 0.02) as important factors for RTW. In this model, resection was positively associated with RTW (as opposed to biopsy with OR, 2.52, 95% CI 1.10–5.80, *p* = 0.03). For this model, the C-statistic was 0.83.

## Discussion

In this nationwide register-based study, the proportion of patients with LGG working 1 year prior to index date was comparable to a matched control group. After 1 and 2 years, respectively, 52% and 63% of the patients were working, while proportions were nearly constant for controls. Our study further provides new insights on predictors affecting RTW in patients with LGG, such as female sex and previous sick leave.

The increase in the proportion of sick leave in patients with LGG started at approximately 6 months prior to surgery, with an exponential increase 3 months prior to surgery. This corresponds well with the fact that for approximately 75% of the patients the time span from initial imaging to surgery was just below 3 months. This indicates a silent period regarding symptoms, and that patients generally work until radiologic diagnosis. However, another Swedish study demonstrated that around 30% of patients with LGG had adjusted workload or tasks 1 year prior to tumor diagnosis.^26^ Thus subtle changes may precede the radiologic diagnosis, but do not necessarily lead to absence from work.

Brain tumor survivors experience limitations at work, due to a higher prevalence of both physical and mental deficits related to the disease itself and to the received treatment.^27^ A few smaller studies present data on RTW in patients with brain tumors including LGG, but there are no larger studies. Instead, studies tend to vary regarding characteristics and prognosis and focus more on specific surgical techniques.^16,17,27^ Comparison with these studies is of limited value since potential factors possibly affecting RTW, as we also demonstrate, will go unnoticed in smaller studies of highly selected patients. In a study on long-term progression-free survivors with anaplastic oligodendrogliomas and oligoastrocytomas, RTW after 2.5 years following diagnosis was 41%.^28^ In contrast, the proportion of RTW in patients with glioblastoma was only 13.8% among patients who had survived the first year.^14^ As expected from tumor biology and differences in age, our data compare favorably, although we included deceased patients in our analyses and counted them as not working.

In the present study, a history of sick leave was a prominent factor affecting RTW, but did not seem to be related to mental health issues, which as suggested in previous studies.^29^ Studies on patient groups of similar ages but with different types of cancer have confirmed the strong association between previous sick leave and no RTW.^30^ This was also the situation for noncancerous conditions affecting the brain.^31^

The fact that lower functional level and more comorbidity were associated with lower rate of RTW is intuitive, and similar findings have been described in patients with glioblastomas.^14^ While comorbidity as defined in our study is independent of the LGG (e.g., seizures excluded), the functional level may be related to the disease itself. We speculate that the strong association between functional level and RTW comes from the fact that lower function per se may inhibit RTW, but also that patients with lower functional level are at risk for shorter survival.^32,33^ With presumed worse prognosis, these patients may be treated differently, with limited focus on rehabilitation but more focus on oncologic treatment. Since we are not only studying patients alive, but define death as no RTW, the impaired survival with lower functional status may affect the findings, especially at 2 years.

Older age has been found to be a negative factor in relation to RTW in several conditions.^25,34-36^ Multiple factors may play a role, such as prolonged convalescence with increasing age, but possibly also a better economic situation and a different attitude towards work. Of note, when including treatment variables in the models, age was no longer an independent predictor. Elderly more often have more aggressive tumor subtypes, with consequences for treatment and prognosis, and these variables might therefore overshadow age in the models.^37^

We also found female sex to be a negative predictor for RTW after 2 years, but not at 1 year. Other factors than sex seem to contribute relatively more initially, but baseline factors may play increasingly larger roles over time. This finding is in line with previous studies where more women than men in general received sickness benefit.^38-40^ However, there are contradicting findings concerning the impact of sex for RTW in other conditions.^13,36,41–44^

Resection (as opposed to biopsy) was shown to be an independent favorable factor for RTW 2 years following surgery. This may, at least to some extent, be explained by selection bias, since patients undergoing resection usually present with more accessible tumors. Nevertheless, it provides a hint that functional outcome is acceptable following surgery where the goal is to prolong life, while preserving social and professional life.^45^ In the case of extensive resection, postponement of adjuvant treatment may be beneficial in terms of RTW in the shorter term since oncologic treatment can obstruct patients' ability to work, especially during treatment.

Studies in other conditions have shown that socioeconomic factors such as income and educational level are important for RTW.^15,34,35^ Although we had no information about type of work, we included disposable income and level of education in our analyses and found that these factors were not independent predictors for RTW.

Differences in social security systems might affect RTW.^46^ In Dutch patients with breast cancer, the proportion with RTW 1 year following diagnosis differed between 43% and 52%, dependent on changes in disability policy.^47^ Thus, RTW in our Swedish cohort with a generous social insurance system might occur more slowly and thus not be directly comparable to the situation in other countries. On the other hand, the Swedish social security system offers both full and partial absence. To exemplify, an American study of patients with breast cancer demonstrated that the proportion of patients who returned to work was 82% (not specified at what level) 1 year after diagnosis.^34^ Similarly, a Swedish study revealed that 83% returned to work at some level within 10 months,^48^ indicating that overall RTW may be less affected by the social beneficial system.

Our study has several limitations. Patients may have been on sick leave due to unrelated causes, but with the availability of a large, properly matched control group, we observe the excess absence. Further, patients without registered sick leave the day of surgery were excluded, since these patients were presumed not to be working. However, there may be rare instances of patients in work not registered for sick leave compensation through the SIA. Finally, we lack molecular tumor data known to be related to prognosis and also to symptom burden, treatment intensity, and thereby affecting RTW.^49,50^

The use of a patient registry with high coverage and relevant clinical variables, the link with other national registries including individual data on sick leave and disability compensation, and a matched control group represent major strengths of this study.

In this nationwide Swedish cohort of patients with LGG, patients were comparable to matched controls in terms of work status 1 year prior to index date. From approximately 6 months prior to surgery, the proportion of patients who received sick leave compensation increased rapidly. At 1 year following surgery, more than half of the patients had returned to work. Previous absence from work, older age, and female sex were all shown to be disadvantageous factors in relation to RTW. In addition, treatment was shown to affect RTW during the first 2 years following surgery.

## Glossary

CI: confidence interval
ICD-10: International Classification of Diseases–10
LGG: low-grade glioma
NBHW: National Board of Health and Welfare
NPR: National Patient Registry
OR: odds ratio
RTW: return to work
SBTR: Swedish Brain Tumor Registry
SIA: Social Insurance Agency
